# Identification and evaluation of the inhibitory effect of *Prunella vulgaris* extract on SARS-coronavirus 2 virus entry

**DOI:** 10.1371/journal.pone.0251649

**Published:** 2021-06-09

**Authors:** Zhujun Ao, Mable Chan, Maggie Jing Ouyang, Titus Abiola Olukitibi, Mona Mahmoudi, Darwyn Kobasa, Xiaojian Yao

**Affiliations:** 1 Department of Medical Microbiology and Infectious Diseases, Laboratory of Molecular Human Retrovirology, Rady Faculty of Health Sciences, College of Medicine, University of Manitoba, Winnipeg, Canada; 2 Special Pathogens Program, National Microbiology Laboratory, Public Health Agency of Canada, Winnipeg, Canada; China Academy of Chinese Medical Sciences, CHINA

## Abstract

Until now, antiviral therapeutic agents are still urgently required for treatment or prevention of SARS-coronavirus 2 (SCoV-2) virus infection. In this study, we established a sensitive SCoV-2 Spike glycoprotein (SP), including an SP mutant D614G, pseudotyped HIV-1-based vector system and tested their ability to infect ACE2-expressing cells. Based on this system, we have demonstrated that an aqueous extract from the Natural herb *Prunella vulgaris* (NhPV) displayed potent inhibitory effects on SCoV-2 SP (including SP_G614_ mutant) pseudotyped virus (SCoV-2-SP-PVs) mediated infections. Moreover, we have compared NhPV with another compound, Suramin, for their anti-SARS-CoV-2 activities and the mode of their actions, and found that both NhPV and Suramin are able to directly interrupt SCoV-2–SP binding to its receptor ACE2 and block the viral entry step. Importantly, the inhibitory effects of NhPV and Suramin were confirmed by the wild type SARS-CoV-2 (hCoV-19/Canada/ON-VIDO-01/2020) virus infection in Vero cells. Furthermore, our results also demonstrated that the combination of NhPV/Suramin with an anti-SARS-CoV-2 neutralizing antibody mediated a more potent blocking effect against SCoV2-SP-PVs. Overall, by using SARS-CoV-2 SP-pseudotyped HIV-1-based entry system, we provide strong evidence that NhPV and Suramin have anti-SARS-CoV-2 activity and may be developed as a novel antiviral approach against SARS-CoV-2 infection.

## Introduction

The recent and ongoing outbreak of Coronavirus disease 2019 (COVID-19) has called for serious and urgent global attention [1, 2]. The COVID-19 disease is caused by a newly emerged virus strain of Severe Acute Respiratory Syndrome (SARS) known as SARS-CoV-2 [1]. Although the case fatality ratio (CFR) of COVID-19 can only be detected at the end of the outbreak, an estimated global CFR was calculated to be 5.5–5.7% in March 2020 shockingly more than the seasonal influenza outbreak [3]. While in August 2020, the infection fatality ratio was estimated by WHO to be 0.5–1% [4]. Since the identification of the SARS-CoV-2 sequences [5], extensive efforts worldwide have been focused on developing effective vaccines and antiviral drugs against SARS-CoV-2 with the hope to rapidly and efficiently control this new human coronavirus (CoV) infection.

SARS-CoV-2 belongs to a betacoronavirus subfamily that includes enveloped, large and positive-stranded RNA viruses responsible for causing severe respiratory system, gastrointestinal and neurological symptoms [6–9]. The human CoV was first identified in 1960 and constituted about 30% of the causes of the common cold. Among the identified human CoVs are NL63, 229E, OC43, HKU1, SARS-CoV, the Middle East respiratory syndrome (MERS)-CoV, and SARS-CoV-2 [10, 11]. A recent study has revealed that SARS-CoV-2 was closely related (88% identity) to two SARS-like CoVs that were isolated from bats in 2018 in China, but it was less related to SARS-CoV (79%) and MERS-CoV (about 50%) [12]. The key determinant for the infectivity of SARS-CoV-2 depends on the host specificity with the viral surface-located trimeric spike (S) glycoprotein (SP), which is commonly cleaved by host proteases into an N-terminal S1 subunit and a membrane-embedded C-terminal S2 region [13]. Recent studies revealed that an SP mutation, Aspartic acid (D) changed to Glycine (G) at amino acid position 614, in the S1 domain has been found in high frequency (65% to 70%) in April to May of 2020, that was associated with an increased viral load and significantly higher transmission rate in infected individuals, but no significant change with disease severity [14]. The following studies also suggested that G614 SP mutant pseudotyped retroviruses infected ACE2-expressing cells markedly more efficiently than those with D614 SP [15].

Up till now, several compounds have been tested in numerous clinical trials, including remdesivir, lopinavir, umifenovir, and hydroxychloroquine [16–20]. Moreover, some *in vitro* research suggested that other drugs such as fusion peptide (EK1), anti-inflammatory drugs (such as hormones and other molecules) could be potentially used in the treatment of SARS-CoV-2 disease (reviewed in [21, 22]). However, their safety and efficacy have not been confirmed by clinical trials. Currently, specific antiviral treatment drugs are still not available for SARS-CoV-2 infections [22].

Traditional Natural medicine holds a unique position among all conventional medication because of its usage over hundreds of years of history. Many aqueous extracts of traditional natural medicinal herbs have been proven to have antiviral activities [23], and most of these are generally of low toxicity, cheap and readily accessible. As an easily free and low-cost natural source, they are incredibly valuable as potential new sources for rapid responses against the ongoing COVID-19 pandemic. *Prunella vulgaris*, widely distributed in China, Europe, northwestern Africa and North America, is known as a self-heal herb. Studies have previously found that a water-soluble substance from Natural herb *Prunella vulgaris* (NhPV) exhibits significant antiviral activity against HIV, HSV and Ebola virus [24–27]. However, whether NhPV can block SARS-CoV-2 virus infection is unknown. Another compound, Suramin, has also been previously shown to be a potent inhibitor against HIV [28], while the subsequent studies revealed that its inhibitory effects on HIV replication did not correlate with clinical or immunologic improvement [29, 30]. A previous study observed that Suramin not only substantially reduced viral loads of *chikungunya virus* (CHIKV) in infected mice, but it also ameliorated virus-induced foot lesions in the mice [31]. Recently, Salgado-Benvindo C. *et al*. reported that Suramin can inhibit SARS-CoV-2 infection in cell culture by interfering with early steps of the replication cycle [32].

In this study, we have established a highly sensitive SARS-CoV-2 SP-pseudotyped virus (SCoV-2 SP-PVs) system and investigated the impact of the cytoplasmic tail and a G614 mutant of SP on virus entry ability. We also examined two compounds, NhPV or Suramin, for their blocking activities in the SCoV-2 SP-PVs system and SARS-CoV-2 infection, and the antiviral mechanism of their actions. Furthermore, we investigated the synergistic effect of combining anti-SARS-CoV-2 neutralizing antibody (nAb) with NhPV or Suramin to enhance their anti-SARS-CoV-2 activity. Overall, this study provides evidence for the first time that NhPV, an aqueous extract from *Prunella vulgaris*, has potent anti- SARS-CoV-2 activity.

## Materials & methods

### Plasmid constructs

The SARS-CoV-2 expressing plasmids (pCAGGS-nCoVSP, pCAGGS-nCoVSPΔC and pCAGGS-nCoVSPΔC_G614_) contain SARS-CoV-2 SP transgene (GenBank accession No. MN908947) or corresponding mutated genes for SPΔC and ΔC_G614_. The SPΔC and ΔC_G614_ were generated by mutagenic PCR technique. Primers are following: SPΔC-3’primer, 5_GCAGGTACCTAGAATTTGCAGCAGGATCCAC; D614G-5’, 5_GCTGTTCTTTATCAGGGTGTTAACTGCACAG; D614G-3’, 5_CTGTGCAGTTAACACCCTGATAAAGAACAGC. Mutated genes were cloned into the pCAGGS plasmid, and each mutation was confirmed by sequencing. The HIV vector encoding for Gaussia luciferase gene HIV-1 RT/IN/Env tri-defective proviral plasmid (ΔRI/E/Gluc) and the helper packaging plasmid pCMVΔ8.2 encoding for the HIV Gag-Pol plasmids are described previously [25, 33].

### Cell culture, antibodies and chemicals

The human embryonic kidney cells (HEK293T) and kidney epithelial cells (VeroE6 and Vero cells (ATCC, CCL-81)) from African green monkeys were cultured in Dulbecco’s modified Eagle’s medium (HEK293T, VeroE6) or Minimum Essential Medium (MEM.; Vero). HEK293T expressing ACE2 (293T_ACE2_) was obtained from GeneCopoeia Inc, Rockville, MD. All cell lines were supplemented with 10% fetal bovine serum (F.B.S.), 1X L-Glutamine and 1% penicillin and streptomycin. The rabbit polyclonal antibody against SARS-CoV-2 SP (Cat# 40150-R007) and ACE2 protein (Cat# 40592-T62) were obtained from Sino Biological and anti-HIVp24 monoclonal antibody was described previously [34, 35]. The HIV-1 p24 ELISA Kit was obtained from the AIDS Vaccine Program of the Frederick Cancer Research and Development Center. SARS-CoV-2 SP-ACE2 binding ELISA kit (Cat# COV-SACE2-1) was purchased from RayBio. Anti-SARS-CoV-2 neutralizing Antibody (nAb) Human IgG1(SAD-535) was purchased from ACRO Biosystems. AH0109 was described previously [36].

### Preparation and purification of herb extracts of Natural herb *Prunella vulgaris* (NhPV)

The dried fruitspikes of *Prunella vulgaris* (Fig 3A) were first soaked overnight in deionized water at room temperature and then boiled for one hour. Then the cooled supernatant was centrifuged (3000 g, 30 min), filtered through a 0.45 μm cellulose acetate membrane and finally lyophilized, as described previously [24]. The resulting dark brown residue was dissolved in deionized water and stored at -20°C. A single symmetrical peak corresponding to a molecular weight of polysaccharides (approximately 10 kDa) in the aqueous extract from NhPV was detected by HPLC analysis, as described previously [24]. Suramin (Cat# sc-200833) was purchased from Santa Cruz BioTech and was dissolved in sterile H2O and stored at -20°.

### Virus production, infection and inhibition experiments

SARS-CoV-2 SP or S.P. ΔC pseudotyped viruses (SCoV-2-SP-PVs, SCoV-2-SPΔC-PVs, SCoV-2-SPΔC_G614_-PVs) were produced by transfecting HEK293T cells with pCAGGS-SARS-CoV-2-SP, pCAGGS-SARS-CoV-2-SPΔC, or pCAGGS-SARS-CoV-2-SPΔC_G614_, pCMVΔ8.2 and a Gluc expressing HIV vector ΔRI/E/Gluc. After 48 hrs of transfection, cell culture supernatants were collected and pseudotyped VLP.s were purified from the supernatant by ultracentrifugation (32,000 rpm) for 2 hrs. The pelleted VPs were resuspended into RPMI medium and virus titers were quantified using an HIV-1 p24 ELISA assay. The SARS-CoV SP-pseudotyped ^Luc+^MLV was produced as described previously [37]. Briefly, 293T cells were transfected with a SARS-CoV S.P. expressing plasmids (pcDNA-SARS-S), pCMV-MLVgagpol MLV plasmid, and pTG-Luc transfer vector. After 48 hrs of transfection, the supernatants were harvested and ready for infection experiments. The wild type SARS-CoV-2 (hCoV-19/Canada/ON-VIDO-01/2020, GISAID accession# EPI_ISL_425177) was propagated and produced in Vero cells (ATCC, CCL-81).

To investigate the infection ability of SCoV-2-SP-VPs, the same amount of each SCoV-2-SP-PV stock (as adjusted by p24 levels) were used to infect different target cells at 0.4 x 10^5^ cells per well (24 well plate) for 3 hrs and washed. After 48 or 72 hrs, the supernatants were collected and the viral infection rate was evaluated by measuring Gaussia luciferase (Gluc) activity. Briefly, 50ul of Coelenterazine substrate (Nanolight Technology) was added to 20ul of supernatant, mixed well and read in the luminometer (Promega, U.S.A.).

To evaluate the anti-SARS-CoV-2 SP-mediated entry activity of NhPV or Suramin, various concentrations of herb extract or compound were directly added into target cells at different time points before or after infection, as indicated. After 3hrs of infection at 37°C, the cells were washed once to remove excessive residue viruses/compound and cultured in fresh medium. The anti-SARS-CoV-2 effects of NhPV or Suramin were evaluated by measuring the Gluc activity or p24 levels in the supernatant infected cultures.

Efficacy of NhPV or Suramin against SARS-CoV-2 (hCoV- 19/Canada/ON-VIDO-01/2020, GISAID accession# EPI_ISL_425177) was evaluated in Vero cells. The Vero cells were seeded into 96-well plates and reached a confluency of 90% at the second day. Then each compound was diluted in assay medium (DMEM with 1X penicillin-streptomycin) and added to the wells (100 ul/well), followed by adding 100 μL of SARS-CoV-2 at a MOI of 0.01, resulting in a final 1X drug concentrations. As positive controls, wells without drugs were infected with SARS-CoV-2 at the same MOI. After 4 hrs of infection, cells were washed and fresh medium (MEM containing 1% FBS., L-Glutamine, and penicillin-streptomycin and the same concentrations of compound) was added to the cells. Cytopathic effect (C.P.E.) was monitored in each well at 48 to 72 hours post-infection (hpi). Viral replication levels were titrated by TCID_50_ assay using supernatants collected at 48hpi. Supernatants were serially diluted 1:10, 100 μL of each dilution was added to 96-well plates of Vero cells in triplicate, plates were incubated at 37°C with 5% CO_2_ and the presence of CPE was determined 4 days pi. The TCID50/mL titer was determined using the Reed and Muench method.

### COVID-19 spike protein-ACE2 binding assay

The inhibitory effect of NhPV or Suramin on the interaction of SP-ACE2 was tested with COVID-19 Spike-ACE2 binding assay kit. Briefly, 96-well plate was coated with recombinant SARS-CoV-2 Spike protein. NhPV or Suramin was then added to the wells for 10 min followed by adding recombinant human ACE2 protein. After incubation for 3 hours, wells were washed three times and a goat anti-ACE2 antibody that binds to the Spike-ACE2 complex was added followed by applying the HRP-conjugated anti-goat IgG and 3,3’,5,5’-tetramethylbenzidine (TMB.) substrate. The intensity of the yellow color is then measured at 450 nm.

### Western blot (WB) analyses

To detect cellular protein ACE2, SARS-CoV-2-SP, or SPΔC in transfected cells or SCoV-2-SP-VPs, transfected 293T_ACE2_ cells or VPs were lysed in RIPA buffer, and directly loaded into the 10% SDS–PAGE gel and the presence of each protein was detected by WB with various corresponding antibodies.

### Cytotoxicity assay

The trypan blue assay was used to determine the effect of NhPV or Suramin on the cell viability and cytotoxicity. Briefly, Vero E6 or 293T_ACE2_ cells were cultured at a density of 1–1.5×10^4^ cells/well in a 96-well format in the presence of various concentrations of NhPV or Suramin for 3hrs, then cells were washed and added with fresh medium. At 48 hours, the cell Viability was tested by trypan blue assay.

### Statistical analysis

Statistical analysis of the infection levels of SARS-CoV-2-SP, or SPΔC-VPs and the inhibition effect of NhPV or Suramin were performed using the unpaired t-test (considered significant at *P*≤0.05) by GraphPad Prism 6.01 software.

## Results

### Generation of a SARS-CoV-2 SP-pseudotyped HIV-1-based entry system

**Fig 1:**
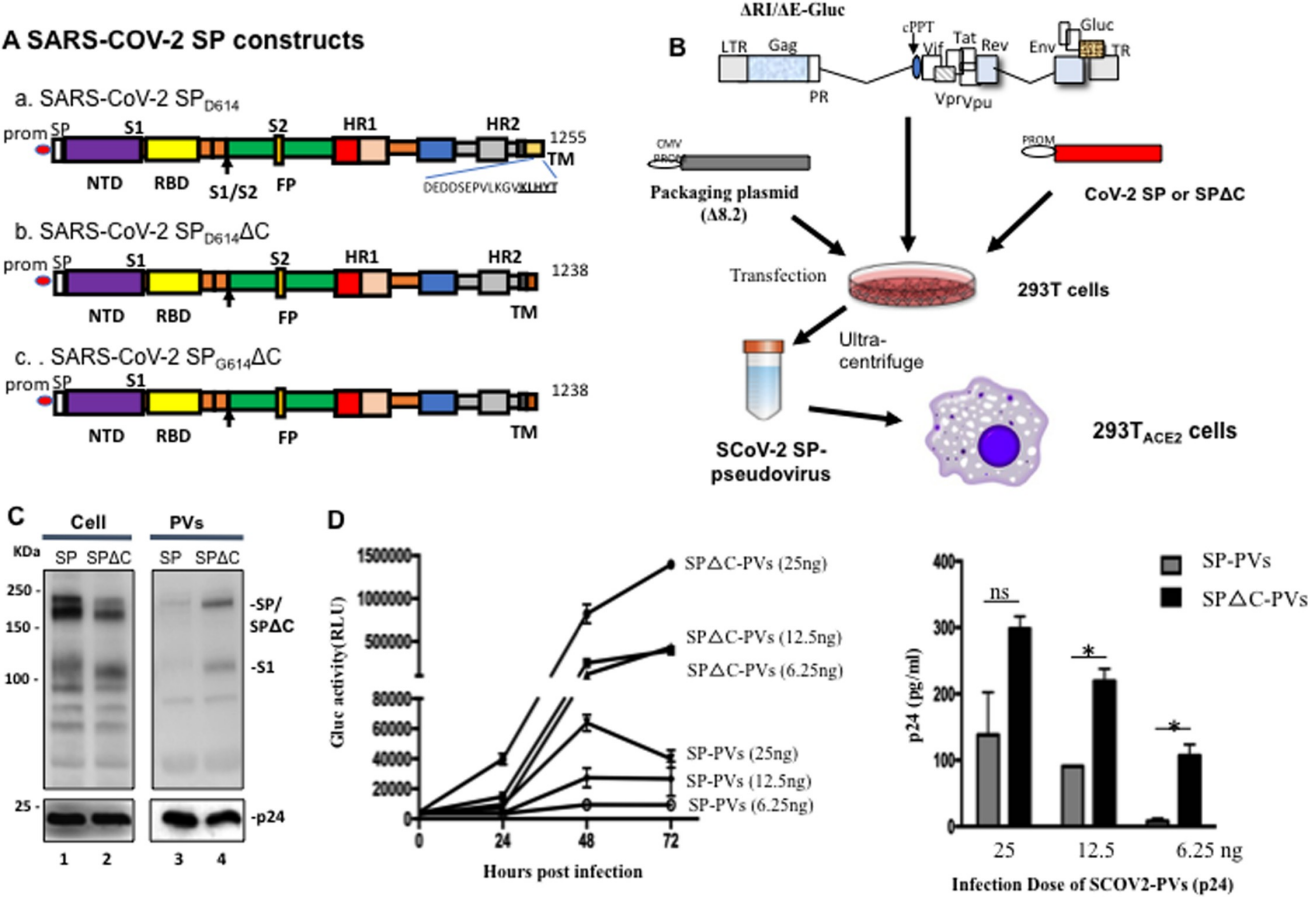
Generation of SARS-CoV2-SP-pseudotyped lentivirus particles (SCoV-2-SP-PVs). A) Schematic representation of SARS-CoV-2SP, SARS-CoV-2SPΔC, and SARS-CoV-2SP_G614_ΔC expressing plasmids. B) Schematic representation of plasmids and procedures for production of SARS-CoV2-SP-pseudotyped lentivirus particles (SCoV-2-SP-PVs). C) Detection of SARS-CoV-2 SPs and HIV p24 protein expression in transfected 293T cells and viral particles by WB with anti-SP or anti-p24 antibodies. D) Different amounts of SCoV-2-SP-PVs and SCoV-2-SPΔC-PVs virions (adjusted by p24) were used to infect 293T_ACE2_ cells. At different time intervals, the Gaussia Luciferase activity (Gluc) (left panel) and PVs-associated p24 (at 72 hrs) in supernatants was measured. Statistical significance was determined using unpaired t-test, and significant *p* values are represented with asterisks, *≤0.05.

Since previous studies have reported that a carboxyl-terminal truncation of 17 amino acids of SARS SP substantially increased SARS SP-mediated cell-to-cell fusion [38], we have constructed a full length SP (SARS-CoV-2-SP) and the C-terminal 17 amino acid (aa) deletion SP (SARS-CoV-2-SPΔC) expressing plasmids (Fig 1A(a, b)). The SARS-CoV-2-SP-mediated virus entry system was established by co-transfecting SARS-CoV-2 SP or SARS-CoV-2-SPΔC expressing plasmid(s), a HIV-based vector (ΔRI/ΔEnv/Gluc) in which viral reverse transcriptase/integrase deleted/envelope gene partially deleted and encoded a Gaussia luciferase gene in the *nef* position [33], and a packaging plasmid (pCMVΔR8.2) in HEK293T cells (Fig 1B). The Gaussia luciferase (Gluc) is a bioluminescent enzyme that can be secreted into the media, enabling the analysis of viral expression by direct measurement of Gluc activity in the supernatant.

The expression and incorporation of SARS-CoV-2 SPs or SARS-CoV-2 SPΔC in the cells and the pseudotyped viruses (SP-PVs, or SPΔC-PVs), were analyzed by SDS-PAGE and Western blot (WB) with a mouse anti-SP antibody, as indicated in Fig 1C. As expected, the HIV capsid Gagp24 protein was detected in all of the cell lysates and the pelleted SP-PVs and SPΔC-PVs by rabbit anti-p24 antibodies (Fig 1C, lower panel). The SARS-CoV-2 SP including S1/S2 was clearly detected in both SARS-CoV-2-SPs and SARS-CoV-2-SPΔC-transfected cells (Fig 1C, lanes 1 and 2). Interestingly our results revealed that virus-incorporation level of SARS-CoV-2 SPΔC were significantly higher than that of SARS-CoV-2-SP (Fig 1C, compare lane 4 to 3).

To test the infectivity of generated pseudoviruses, we infected 293T-ACE2 cells with serial diluted amounts (25, 12.5, 6.25ng of p24) of SP-PVs or SPΔC-PVs for 3 hrs. The Gluc activities or Gagp24 of supernatants from infected cells were measured at 24h, 48h or 72h post infection. The results showed that both SP-PVs and SPΔC-PVs infected 293T-ACE2 cells and induced an increase of Gluc activity in the supernatants in a dose dependent manner (Fig 1D, left panel). As expected, the infectivity of SPΔC-PVs was significantly higher than that of SP-PVs. The infection of pseudoviruses in 293T_ACE2_ cells was further confirmed by detection of the HIVp24 levels in the supernatants of infected cells through ELISA assay (Fig 1D, right panel).

**Fig 2:**
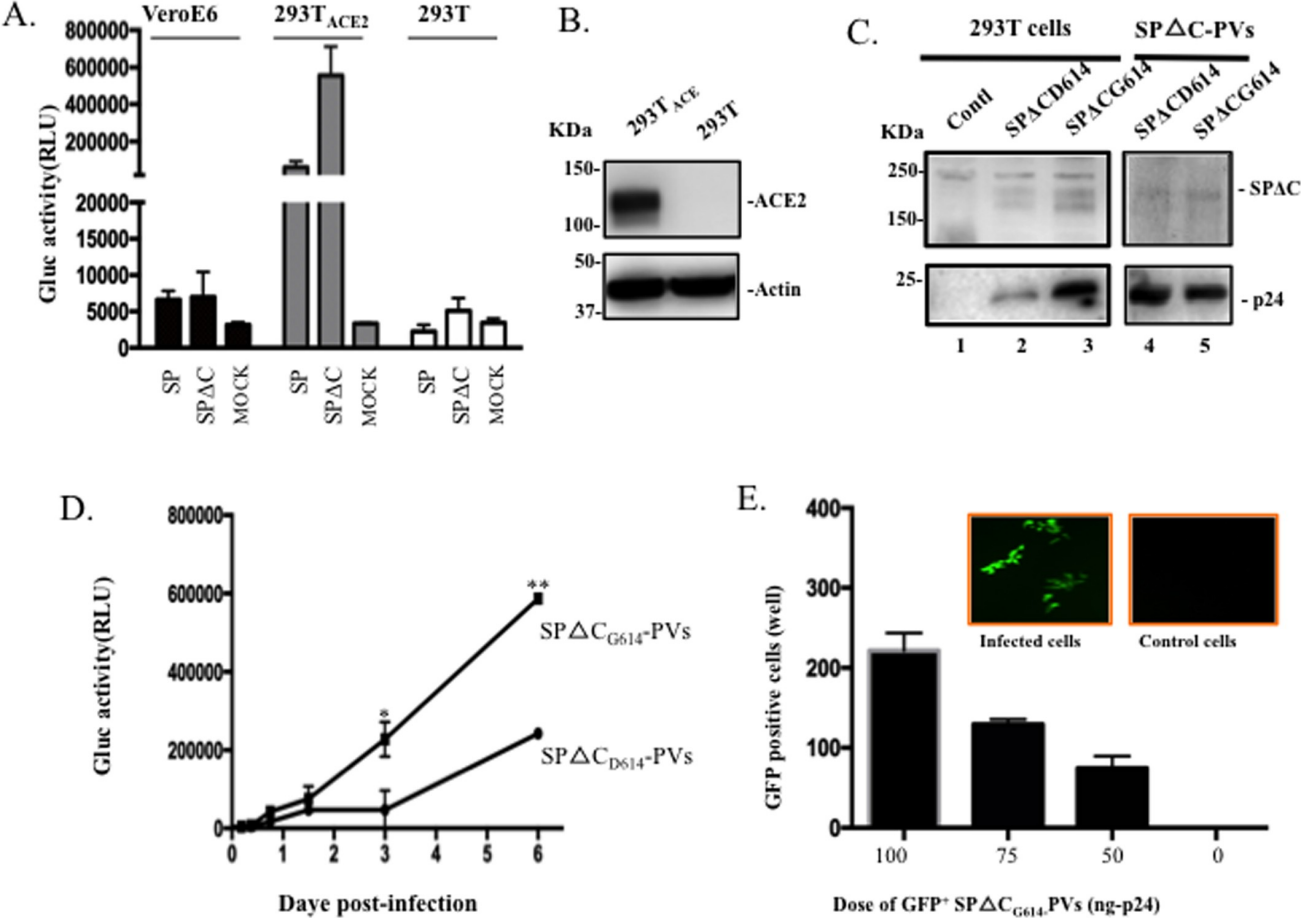
SARS-CoV-2 SP-PVs’s infection in different cell lines and SARS-CoV-2 SP_G416_ variant exhibited stronger virus entry. A) 293T_ACE2,_ 293T, and Vero-E6 cells were infected by equal amounts of SARS-CoV-2SP- or SARS-CoV-2SPΔC-pseudotyped viruses (PVs), or mock-infected (MOCK). At 48 hrs pi, the Gluc activity in supernatants was measured. B) The expression of SARS-CoV-2SP receptor, ACE2 in 293T and 293T_ACE2_ cells detected by WB with anti-ACE2 antibodies. C) Detection of SARS-CoV-2 SPΔC, SPΔC_G614_ and HIV p24 protein expressions in transfected 293T cells and viral particles by WB with corresponding antibodies. D) Infectivity comparison of SPΔC _D614_-PVs and SPΔC_G614_-PVs in 293T_ACE2_ cells. Equal amounts of SPΔC_D614_-PVs and SPΔC_G614_-PVs virions (adjusted by p24 level) were used to infect 293T_ACE2_ cells. At different days post-infection (pi), Gluc activity in supernatants was measured. The results are the mean ±SD of duplicate samples, and the data are representative of results obtained in two independent experiments. E) The SPΔC_G614_-GFP^+^PVs were produced from 293T cells and used to infect 293T_ACE2_ cells in 96-well plate After 48 hrs pi, GFP-positive cells (per well) were counted and photographed by fluorescence microscope (on the top of the panel). Statistical significance was determined using unpaired t-test, and significant *p* values are represented with asterisks, *≤0.05, **≤0.01.

To test whether the infection is ACE2-dependent, we infected various cell lines, including HEK293T, 293T_ACE2_ and VeroE6 with SP-PVs and SPΔC-PVs, respectively. The results showed that these pseudoviruses was able to efficiently infect 293T_ACE2_ cells, has lower level of infection in VeroE6 cells, but could not infect HEK293T (Fig 2A). In parallel, we detected high level expression of the SARS-CoV-2 receptor ACE2 in 293T_ACE2_ cells (Fig 2B).

### SARS-CoV-2 SP G416 variant exhibited more efficient virus entry

Recent sequence analyses revealed a SP mutation, Aspartic acid (D) changed to Glycine (G) at aa position 614, was found in high frequency (65% to 70%) in April to May of 2020, indicating a transmission advantage to D614 [14]. In this study, we have also generated constructs to express SCoV-2-SPΔC_G614_ (Fig 1A(c)) and compared its virus entry ability with SCoV-2-SPΔC (SPΔC_D614_). Our results showed that SCoV-2-SPΔC_G614_ was incorporated into viruses similar to SCoV-2-SPΔC_D614_ (Fig 2C, compare lane 5 to lane 4), however, the SARS-CoV2-SPΔC_G614_-pseudotyped viral particles (SPΔC_G614_-PVs) mediated approximately 3-fold higher infection than that of SPΔC_D614_-PVs (Fig 2D), suggesting that the SP_G614_ mutation increases SP-mediated viral entry.

In addition, we have generated a GFP^+^ SARS-CoV-2-SP-mediated virus entry system by co-transfecting SARS-CoV-2 SPΔC_G614_, a lentiviral vector that expressing GFP, and the pCMVΔR8.2 in HEK293T cells and produced SPΔC_G614_-PVs expressing GFP (GFP^+^ SPΔC_G614_-PVs). After 293T_ACE2_ cells were infected with GFP^+^ SPΔC_G614_-PVs, the GFP positive 293T_ACE2_ cells were clearly detected under fluorescent microscopy (Fig 2E). All of data indicates that both Gluc+, GFP-expressed SARS-CoV-2-SP-pseudotyped virus infection systems can be very sensitive system for studying the functions of SARS-CoV-2-SP variants.

### Evaluation of NhPV and Suramin for blocking SARS-CoV2-SP-mediated virus entry

**Fig 3:**
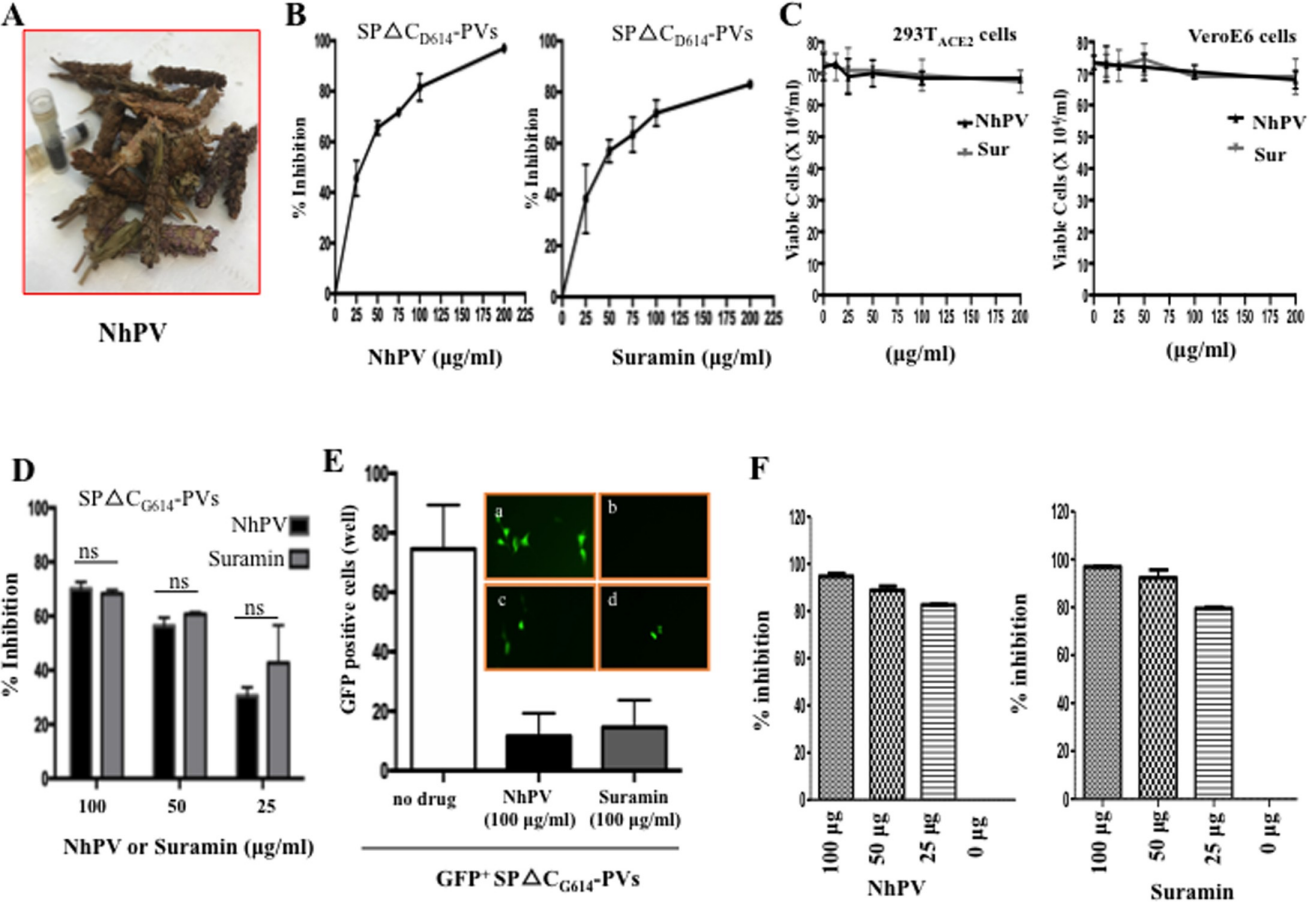
Both SARS-CoV-2 SP- and SARS-CoV SPΔC_G614_-PV’s infection was efficiently blocked by NhPV and Suramin. A) Images of the dried Prunella Vulgaris flowers and its water extract (NhPV). B) The analysis of anti-SARS-CoV-2 activities of NhPV or Suramin. 293T_ACE2_ cells were infected by equal amounts of SARS-CoV-2SPΔC-pseudotyped viruses in the presence of different dose of NhPV or Suramin. At 48 hrs pi, the Gluc activity in supernatants was measured. (% inhibition = 100 x [1 - (Gluc value in presence of drug)/(Gluc value in absence of drug)). C) The cytotoxicity analysis of NhPV and Suramin in 293T_ACE2_ cells and VeroE6 cells. 293T_ACE2_ cells or VeroE6 cells in 96 well plates were treated with different concentration of NhPV or Suramin for 3hrs, compounds were washed and replaced by fresh medium. At 48 hours, the cell Viability was tested by trypan blue assay. D) Infection inhibition of NhPV or Suramin on SARS-CoV-2-SPΔC_G614_-PVs in 293T_ACE2_ cells. Equal amounts of SCoV-2-SPΔC_G614_-PVs (adjusted by p24 level) were used to infect 293T_ACE2_ cells in presence of different concentrations of NhPV or Suramin, in indicated at bottom of the panel. At 48 hrs pi, Gluc activity in supernatants was measured and present as % inhibition. Means ±S.D. were calculated from duplicate experiments. E) 293T_ACE2_ cells in 96-well plate were infected with SP△C_G614_-GFP^+^ PVs. After 48 hrs pi, GFP-positive cells (per well) were counted (left panel) and photographed by fluorescence microscope (top panel, a. Without drugs; b. Without infection; c. In the presence of NhPV (100 μg/ml**)**; d. In the presence of Suramin (100 μg/ml**)**. F) 293T_ACE2_ cells in 96-well plate were infected with SARS-CoV SP-pseudotyped ^Luc+^ MLV. At 48 hrs pi, the infected cells were lysed and cell-associated luc activity was measured and calculated as % inhibition (% inhibition = 100 x [1 - (Luc value in presence of drug)/(Luc value in absence of drug)). The results are the mean ±SD of duplicate samples, and the data are representative of results obtained in two independent experiments.

Next we tested whether NhPV (Fig 3A) and Suramin could block SARS-CoV2 SP-mediated virus entry of 293T_ACE2_ cells since both NhPV and Suramin have been previously reported to exhibit the antiviral activities against several viral infections [24–28, 31]. Briefly, 293T_ACE2_ cells were infected with SPΔC-PVs in the presence of different concentrations (25, 50, 75, 100 and 200ug/ml) of NhPV (Fig 3B, left panel) or Suramin (Fig 3B, right panel), respectively. After 3 hour of infection, the infected cells were washed to remove the viruses and compounds and cultured with fresh medium. At 48 hrs post-infection, the supernatants were collected and the virus-produced Gluc activities were measured for monitoring the infection levels. Consistent with our previous observation [25], we did not detect any toxic effect on either 293T_ACE2_ cells or Vero E6 cells for 3hs exposure to NhPV, nor to Suramin (Fig 3C). Significantly, both NhPV and Suramin were able to inhibit SARS-CoV-2-SP-pseudotyped virus infection. The half maximal inhibitory concentration (IC50) of NhPV was 30 ug/ml (Fig 3B, left panel), while IC50 of Suramin was about 40 ug/ml (Fig 3B, right panel). The inhibitory effect of NhPV and Suramin on a SP mutant pseudotyped virus (SPΔC_G614_-PVs) infection was also tested, and results show that SPΔC_G614_-PVs infection is also susceptible to NhPV and suramin (Fig 3D). Meanwhile, the SARS-CoV-2-SPΔC_G614_ pseudotyped GFP+ virus infection was monitored in the presence of NhPV or Suramin and results showed that the pseudotyped GFP+ virus infection was efficiently inhibited by the presence of NhPV and Suramin (Fig 3E). Furthermore, we also tested the inhibitory effects of NhPV and Suramin on severe acute respiratory syndrome coronavirus (SARS-CoV) spike protein-mediated infection by using a SARS-CoV SP-pseudotyped MLV infection system, as described previously [37]. Results showed that both NhPV and Suramin block SARS-CoV SP-mediated infection in 293T-ACE2 cells (Fig 3F). All of these results demonstrate that both NhPV and Suramin exhibit strong inhibitory effect on infections mediated by SPΔC_D614_-PVs, SPΔC_G614_-PVs and SARS-CoV SP-PVs.

### Mechanistic analyses of actions of NhPV and Suramin against SARS-CoV-2-SP-mediated virus entry

**Fig 4:**
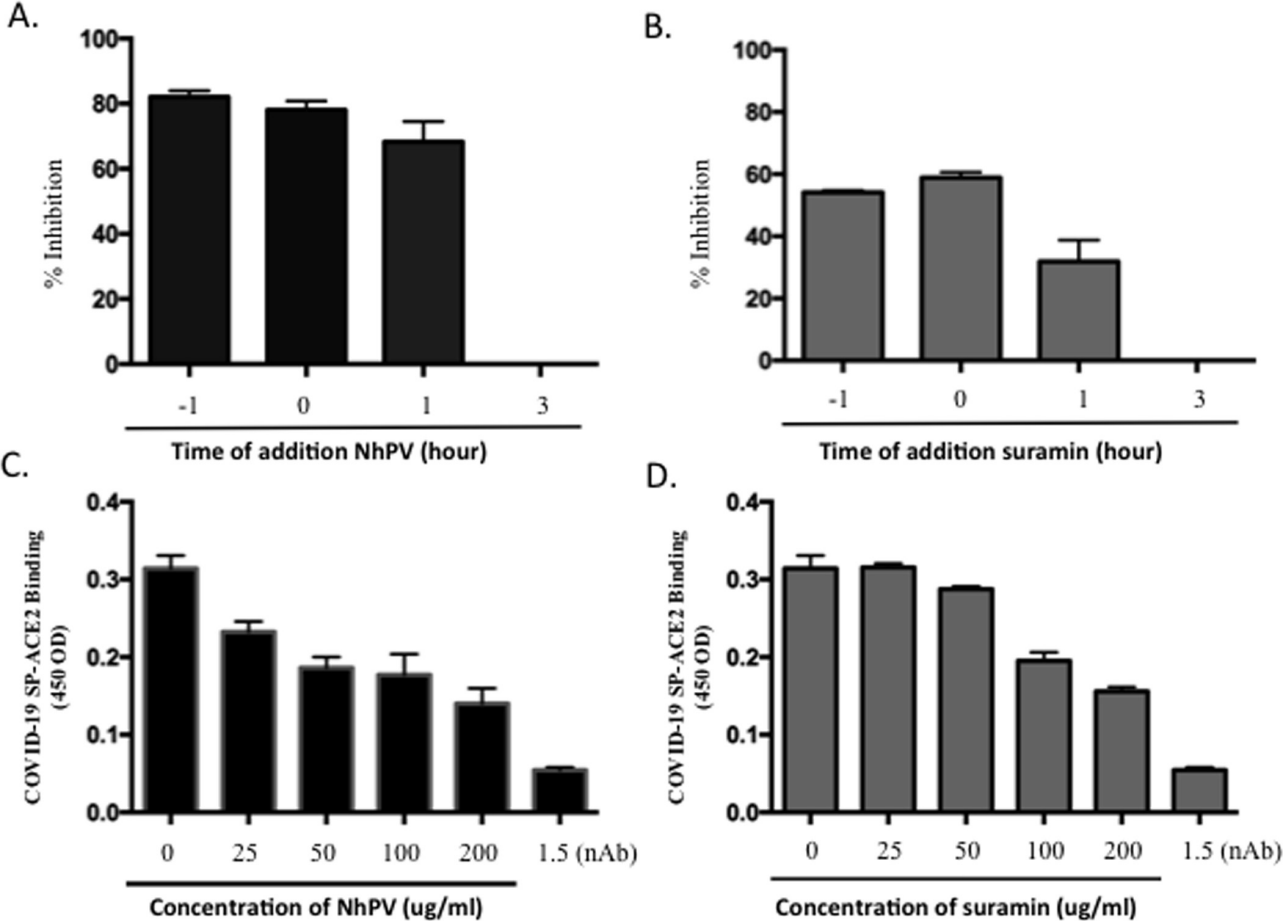
Characterization of the mechanisms of NhPV and Suramin for their anti-SARS-CoV-2-SP action. A) Time-dependent inhibition of SPΔC_G614_-PVs infection mediated by NhPV or Suramin. NhPV (100 μg/mL) or Suramin (100 μg/mL) was added at 1 hr prior to infection, during infection (0 hr), and at 1 hr, and 3 hr pi. The positive controls (P.C.) were 293T_ACE2_ cells infected with SPΔC_G614_-PVs in the absence of compounds. At 3 hrs pi, all of the cell cultures were replaced with fresh DMEM and cultured for 48 hrs. Then, the Gluc activity was monitored in the supernatant, and the data are shown as a percentage of inhibition (%). B) Inhibitory effect of NhPV or Suramin on SARS-CoV2-SP/ACE2 binding by ELISA as described in materials and methods. nAB: anti-COVID-19 neutralizing antibody (SAD-S35). The results are the mean ±S.D. of duplicate samples, and the data are representative of results obtained in two independent experiments.

To gain more insight into the mechanism of how NhPV or Suramin targets SARS-CoV-2 SP-PVs infection, each of the drugs (100 μg/ml) was added to 293T_ACE2_ cells at various time points during the infection, as indicated in Fig 4. After 48 hrs of infection, the supernatants were collected and measured for virus-expressed GLuc activity. Results showed that a strong inhibitory effect was achieved when cells were pretreated with NhPV or Suramin one hour before infection or when the compounds were present simultaneously with SPΔC_G614_-PVs (Fig 4A and 4B). Interestingly, even when drug was added at one hour post-infection, NhPV still exhibited nearly 70% inhibition on SPΔC_G614_*-*PVs infection (Fig 4A), while for Suramin, a lower inhibitory effect (about 30% inhibition) was also observed (Fig 4B). When NhPV or Suramin was added to culture after 3 hrs of infection, no inhibitory activity on viral infection was observed (Fig 4A and 4B). These results suggest that both NhPV and Suramin act on the entry step of SPΔC_G614_*-*PVs infection.

To further determine whether NhPV or Suramin is targeting the interaction of SARS-CoV2-SP and its receptor, ACE2, we used an *in vitro* SARS-CoV2-SP/ACE2 binding ELISA assay, as described in Materials and methods. Additionally, an anti-COVID-19 neutralizing antibody (SAD-S35) [39] was included in parallel. Results revealed that the presence of either NhPV or Suramin was able to specifically target and significantly reduce the SARS-CoV2-SP-ACE2 interaction (Fig 4C and 4D). It should be noted that the neutralizing antibody (SAD-S35) also showed a strong inhibition on SARS-CoV2-SP/ACE2 interaction (Fig 4C and 4D).

### Combination of NhPV and anti-SARS-CoV-2 neutralizing antibody (SAD-S35) mediated more potent blocking effect against SARS-CoV2-SP-PVs

**Fig 5:**
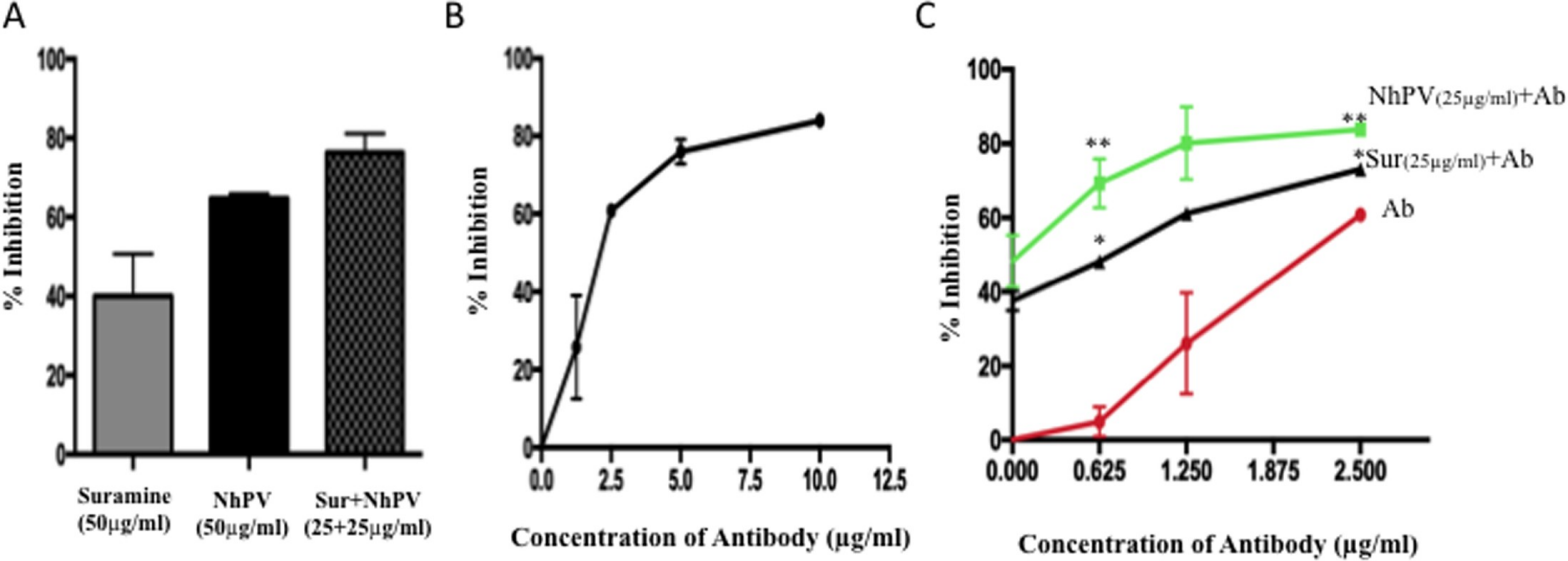
Enhanced inhibitory effects mediated by combination of NhPV and Suramin with neutralizing antibody (SAD-S35). A) 293T_ACE2_ cells were infected with SPΔC_G614_-PVs in presence of NhPV (50μg/ml) or suramin (50μg/ml) alone or a mix of NhPV and suramin (each with 25μg/ml). The Gluc activity in the supernatant was measured after 48 hrs and present as % inhibition. B) Inhibitory effect of nAb SAD-S35 on SPΔC_G614_-PVs infection. 293T_ACE2_ cells were infected with SPΔC_G614_-PVs in the presence of serially diluted SAD-S35 (1.25 to 10 μg/ml) for 3 hrs. Then infected cells were cultured in fresh medium. At 48 hrs pi., the Gluc activities in the supernatants were measured and presented as % inhibition. C) 293T_ACE2_ cells were infected with SPΔC_G614_-PVs in the presence of serially diluted SAD-S35 (0.625 to 2.5 μg/ml) alone or mixed with NhPV (25 μg/mL) or Suramin (25 μg/mL) for 3 hrs and the infected cells were cultured in fresh medium. At 48 hrs pi., the Gluc activities in the supernatants were measured and presented as % inhibition. The results are the mean ± S of duplicate samples, and the data are representative of results obtained in two independent experiments. Statistical significance was determined using unpaired t-test between NhPV+Ab/Ab or Suramin +Ab/Ab, and significant *p* values are represented with asterisks, *≤0.05, **≤0.01.

As described above, both NhPV and Suramin can inhibit SARS-CoV2-SP/ACE2 interaction and SP-PVs infection. We thus tested whether the combination of two compounds could mediate a stronger anti-SARS-CoV-2 activity. 293T_ACE2_ cells were infected with SPΔC_G614_-PVs in the presence of a cocktail of NhPV/Suramin (25 μg/mL per compound), or NhPV (50 μg/mL) or Suramin (50 μg/mL) alone. The results showed that in the presence of a cocktail of NhPV/Suramin, SPΔC_G614_-PVs was inhibited to 78%, while in the presence of NhPV or Suramin alone, inhibition rate was 65% or 40% (Fig 5A). These results suggest that a combination of these two compounds may be able to achieve more efficient inhibition against SARS-CoV-2 infection.

The anti-SARS-CoV-2 neutralizing antibody (SAD-S35) was also tested and showed a does-dependent neutralizing activity against SPΔC_G614_-PVs with an IC50 of 2.4 μg/mL (Fig 5B). Next, we sought to determine whether the combination of NhPV or Suramin with SAD-S35 could mediate a stronger anti-SARS-CoV-2 activity. Thus, serially diluted SAD-S35 (0.625 to 2.5 μg/ml) was mixed with NhPV (25 μg/mL) or Suramin (25 μg/mL) and added to the 293T_ACE2_ cells with SPΔC_G614_-PVs simultaneously. In parallel, the same concentrations of SAD-S35 alone were used for comparison. The results show that 1.25 μg/ml of SAD-S35 alone only resulted in an approximately 25% decrease of infection. However, nearly 80% inhibitory effect was achieved when the same concentration of SAD-S35 was combined with NhPV (25μg/ml), or approximately 60% inhibitory effect was achieved when combined with Suramin (25μg/ml), while NhPV or Suramin alone only mediated approximately 50% or 38% inhibition, respectively (Fig 3B). All together, the results clearly indicate that a combination of NhPV or Suramin with SAD-S35 is able to more potently block SARS-CoV2 infection. By including a low dose of nAb, the amounts of NhPV or Suramin needed to achieve highly effective inhibition of SARS-CoV2 infection can be reduced.

### Inhibitory effect of NhPV and Suramin on SARS-CoV-2 virus infection

**Fig 6:**
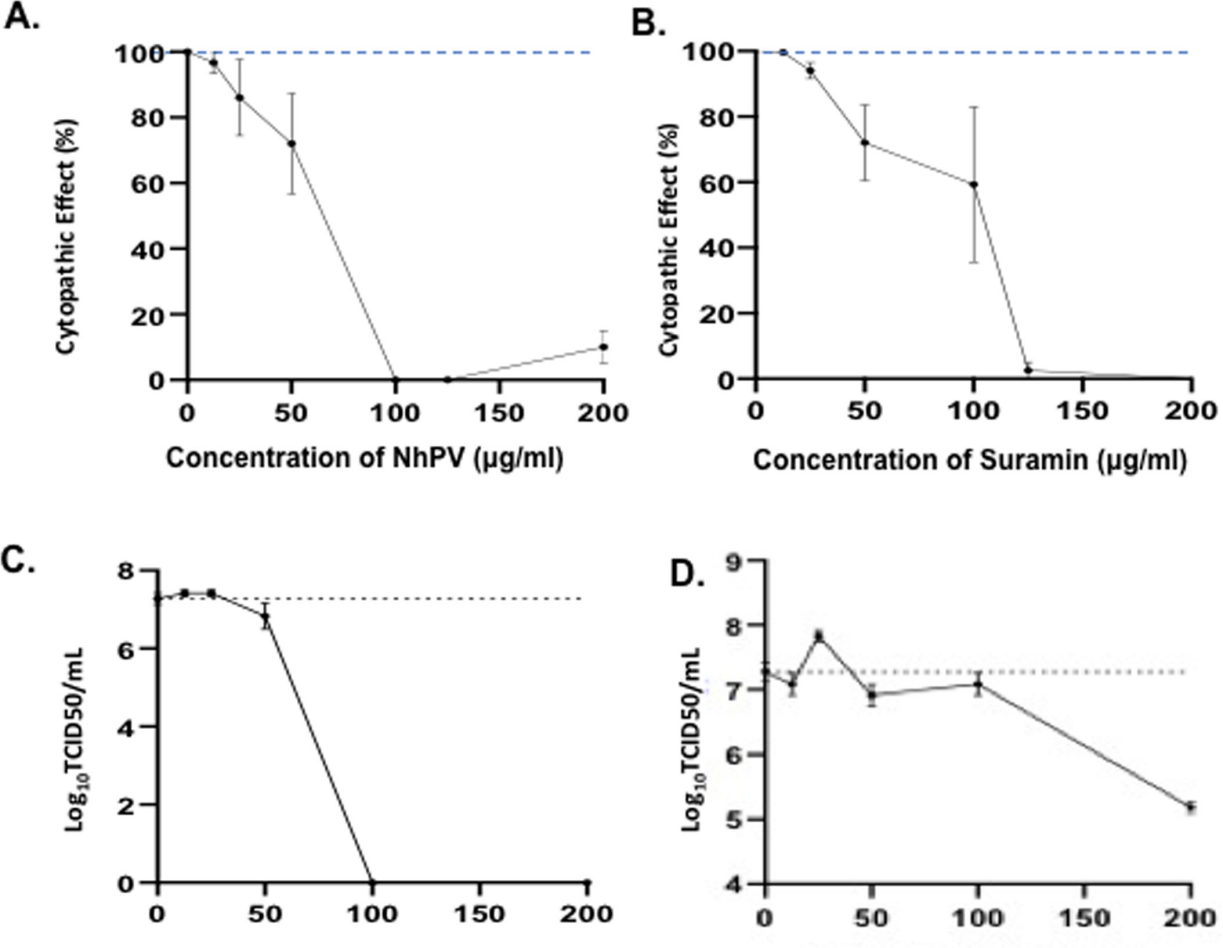
Inhibitory effect of NhPV and Suramin on SARS-CoV-2 infection-induced cytopathic effects. Vero cells were infected with a wild type *SARS-CoV-2* virus (hCoV- 19/Canada/ON-VIDO-01/2020) in the presence or absence of different concentrations of NhPV and Suramin. After 72 hrs pi., the SARS-CoV-2 infection-induced cytopathic effects in Vero cells were monitored. Error bars represent variation between triplicate samples, and the data of (A) and (B) are representative of results obtained in three independent experiments. Also, the supernatants from SARS-CoV-2 infected cultures in the presence of different concentrations of NhPV (C) or Suramin (D) were collected and the levels of progeny virus produced from infected cultures were titrated by TCID_50_ assay in Vero cells. The results are the mean ±S.D. of duplicate samples, and the data are representative of results obtained in two independent experiments.

Given that both NhPV and Suramin are able to block the SARS-CoV2-SP pseudovirus entry, we next tested whether these two drugs could block wild type SARS-CoV2 virus infection and virus-induced cytopathic effect in Vero cells. The wild type SARS-CoV-2 virus (hCoV- 19/Canada/ON-VIDO-01/2020) was used to infect Vero cells in the presence of different concentrations of NhPV or Suramin. Briefly, Vero cells were infected with SARS-CoV-2 (MOI of 0.01) in the presence of different concentrations of NhPV, or Suramin. After 72 hrs post-infection, as indicated in Fig 6, the SARS-CoV-2-induced cytopathic effects in Vero cells were monitored. Results showed that SARS-CoV-2 infection caused dramatic cytopathic effect (CPE) in Vero cells after 72 hrs post-infection, with cells displaying 100% CPE. Remarkably, in the presence of NhPV or Suramin (at 50 to 125 μg/ml), the SARS-CoV-2-induced cytopathic effect (CPE) was partially or completely inhibited in Vero cells (Fig 6A and 6B). Also, we directly monitored the inhibitory effects of NhPV and Suramin on SARS-CoV-2 virus infection by titrating the produced progeny viruses by TCID_50_ assay. Results revealed that at 100 μg/ml, NhPV completely inhibited SARS-CoV-2 virus infection (Fig 6C), while Suramin significantly reduced SARS-CoV-2 virus replication at 200 μg/ml (Fig 6D). These results provide strong evidence that the presence of NhPV or Suramin is able to inhibit SARS-CoV-2 infection.

## Discussion

Because SARS-CoV-2 is classified as an aerosol biosafety level 3 (BSL-3) pathogen, the study of SARS-CoV-2 infection and the investigation of different anti-SARS-CoV-2 compounds required highly restricted BSL-3 containment. This condition has significantly limited the SARS-CoV-2-related research activities in microbiology laboratories. In this study, we established a highly sensitive SARS-CoV-2-SP pseudotyped HIV-based entry system, which encodes a Gaussia luciferase (Gluc) gene as a reporter (Fig 1B). Since Gluc can be secreted into the supernatant after being expressed in the infected cells, it is very sensitive and convenient for evaluating the level of SARS-CoV2-SP-mediated virus entry and may be used for anti-SARS-CoV2-SP compound screening in a BSL-2 environment.

Previous studies have revealed that the cytoplasmic tail (CT) of SARS SP contains a dibasic motif (KxHxx) that constitutes for an endoplasmic reticulum (ER) retrieval signal which retains the full-length SARS-S protein in the lumen of the ER-Golgi intermediate compartment (ERGIC) [40, 41]. Deletion of 17 aa at the carboxyl-terminal in the CT of SARS SP was able to increase SP transported to the surface of cells and substantially increased SARS SP-mediated cell-to-cell fusion [38]. In the SARS-CoV2-SP, there is also a dibasic motif (KxHxx) present in the CT (Fg 1A). In order to increase SARS-CoV2-SP incorporation into pseudovirions, we have deleted 17 aa at the CT of SARS-CoV2 SP and generated a SARS-CoV2 SPΔC expressor plasmid (Fig 1A(b)). Indeed, our data showed that a significantly higher level of SCoV2 SPΔC protein was present in the pseudovirus (Fig 1C), and induced remarkably efficient infection in 293T_ACE2_ cells (Fig 1D). This observation clearly indicates that the dibasic motif in SARS-CoV-2 SP is functional and a deletion of 17 amino acids substantially increased incorporation of SP into SARS-CoV2-SP-PVs and enhance its infectivity. This information is also important for improving the design of SARS-CoV2-SP-based vaccine strategies.

Recent sequencing analyses found a SARS-CoV2 SP mutation, Aspartic acid (D) changed to Glycine (G) at aa position 614 in the S1 domain which was dominantly detected in April to May of 2020 isolates, indicating a transmission advantage over original SP D614 [14]. The following studies showed that SARS-CoV2_G614_ SP mutant MLV pseudotyped viruses infected ACE2-expressing cells markedly more efficiently than those with SARS-CoV2_D614_ [15, 42]. Consistently, we also observed the SARS-CoV2_G614_ΔC-pseudotyped lentiviral particles enhanced the pseudotyped virus entry compared to the SP_D614_ΔC-PVs (Fig 2E).

By using this SCoV2-SP- pseudovirus system, we have provided evidence for the first time that the NhPV can efficiently prevent infections mediated by both SARS-CoV2-SP_D614_ and SARS-CoV2-SP_G614_ pseudovirus infection in 293T_ACE2_ cells (Fig 3) and significantly block the infection of the wild type SARS-CoV-2 in Vero cells (Fig 6). We also revealed that NhPV was able to directly interrupt the interaction of SARS-CoV2-SP and ACE2 receptor by *in vitro* ELISA assay (Fig 4C). Interestingly, the presence of NhPV at one hour post-infection is still able to efficiently inhibit SARS-Cov2-SP pseudovirus infection (Fig 4A), suggesting that NhPV may not only target SP/ACE2 binding, but may also act on the following fusion step(s). Overall, our results provide convincing evidence for NhPV as a potential blocking agent against SARS-CoV-2 infection. In agreement with a recent study [32] that Suramin inhibited SARS-CoV-2 virus infection by acting the early stage, we further provide evidence that Suramin is able to directly block SARS-CoV-2 SP-ACE2 interaction (Fig 4D), inhibit different SARS-CoV-2 SP variants mediated virus entry (Figs 3–5) and SARS-CoV-2 infection induced cytopathic effects (Fig 6B).

Another interesting observation in this study is that the combination of NhPV or Suramin with anti-SARS-CoV-2 neutralizing antibody (nAb) could enhance their anti-SARS-CoV-2 activity. The nAbs have great potential to be used as a preventing agent in blocking SARS-CoV-2 infection [43]. However, one disadvantage of using nAb as an anti-SARS-CoV-2 agent is its source limitation. Therefore, the finding of the synergistic effect of a combination of nAb with other agents, such as NhPV or Suramin is beneficial for (i) similar efficiencies would be achieved by using reduced amounts of antibody and NhPV or Suramin, (ii) the combination of nAb and NhPV/suramin will reduce the likelihood of viral resistance. Whether these enhanced effects might be due to a combined effect through their different binding mechanisms still needs to be investigated.

The effectiveness of NhPV and/or Suramin against SARS-CoV-2 infection *in vivo* remains to be investigated. Our findings could be further validated in an appropriate animal model and clinical trials for prevention of COVID-19. Since SARS-CoV-2 infection initiates in the respiratory tract [44], the use of NhPV and/or Suramin as nasopharynx agents (Nasal spray) to prevent initial SARS-CoV-2 infection and transmission in the respiratory tract will be a particularly attractive strategy, and will require further efficacy studies. Overall, we demonstrated that NhPV and Suramin possess an anti-SARS-CoV-2 entry inhibitor activity and functions at least partially by interrupting SARS-CoV-2 SP binding to its receptor. Additional *in vivo* safety and protection studies will facilitate its application as an option to help control the ongoing SARS-CoV-2 pandemic.

## Supporting information

S1 Raw images (PDF)
