# Supplementary figures and images for: Identification and evaluation of the inhibitory effect of Prunella vulgaris extract on SARS-coronavirus 2 virus entry
Source: PLoS One. 2021 Jun 9;16(6):e0251649. doi: 10.1371/journal.pone.0251649 (PMC8189562; doi:10.1371/journal.pone.0251649)

Fig. 1C, Western blot, lane 1,2,3,4

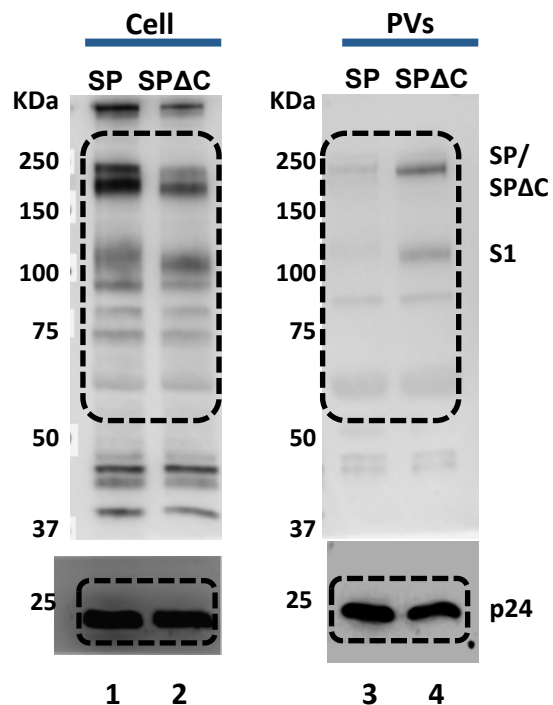

Fig. 2B, Western blot

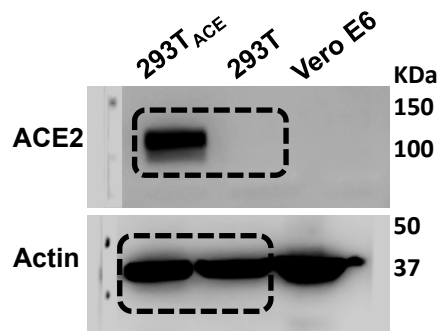

Fig. 2C. Western blot, lane 1,2,3

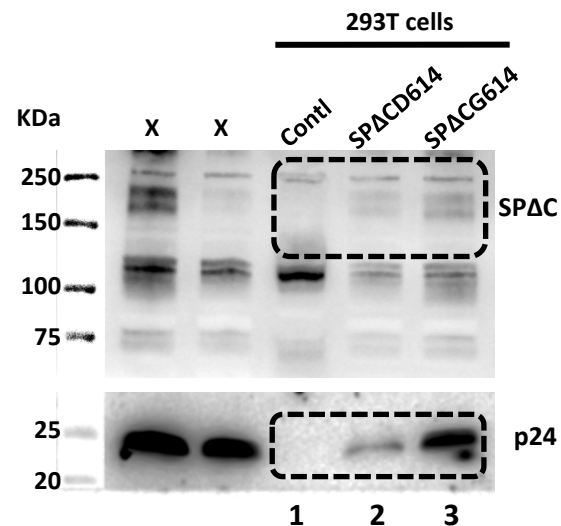

Fig. 2C, Western blot, lane 4 and 5

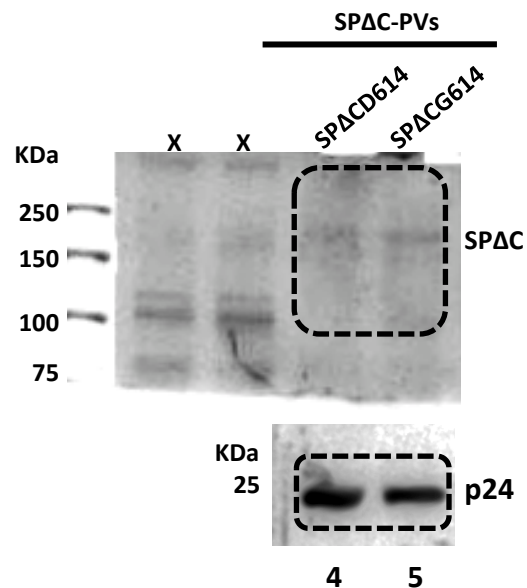

Supplement: S1 Raw images — (PDF) [file pone.0251649.s001.pdf]
